# An inter-country comparison of unofficial payments: results of a health sector social audit in the Baltic States

**DOI:** 10.1186/1472-6963-8-15

**Published:** 2008-01-21

**Authors:** Anne Cockcroft, Neil Andersson, Sergio Paredes-Solís, Dawn Caldwell, Steve Mitchell, Deborah Milne, Serge Merhi, Melissa Roche, Elena Konceviciute, Robert J Ledogar

**Affiliations:** 1 CIETeurope, PO Box 8636, London SW6 2ZB, UK; 2 Centro de Investigación de Enfermedades Tropicales, Universidad Autónoma de Guerrero, Acapulco, México; 3 CIETcanada, 319-1 Stewart Street, Ottawa, K1N 6N5, Canada; 4 CIET Trust, 71 Oxford Road, Saxonwold, Johannesburg, 2196, South Africa; 5 Specialjuju Tyrimu Tarnyba (STT), Vilnius, Lithuania; 6 CIETinternational, 511 Avenue of the Americas, #132, New York, NY 10011, USA

## Abstract

**Background:**

Cross-country comparisons of unofficial payments in the health sector are sparse. In 2002 we conducted a social audit of the health sector of the three Baltic States.

**Methods:**

Some 10,320 household interviews from a stratified, last-stage-random, sample of 30 clusters per country, together with institutional reviews, produced preliminary results. Separate focus groups of service users, nurses and doctors interpreted these findings. Stakeholder workshops in each country discussed the survey and focus group results.

**Results:**

Nearly one half of the respondents did not consider unofficial payments to health workers to be corruption, yet one half (Estonia 43%, Latvia 45%, Lithuania 64%) thought the level of corruption in government health services was high. Very few (Estonia 1%, Latvia 3%, Lithuania 8%) admitted to making unofficial payments in their last contact with the services. Around 14% of household members across the three countries gave gifts in their last contact with government services.

**Conclusion:**

This social audit allowed comparison of perceptions, attitudes and experience regarding unofficial payments in the health services of the three Baltic States. Estonia showed least corruption. Latvia was in the middle. Lithuania evidenced the most unofficial payments, the greatest mistrust towards the system. These findings can serve as a baseline for interventions, and to compare each country's approach to health service reform in relation to unofficial payments.

## Background

Estonia, Latvia and Lithuania reformed their health systems after emerging from the Soviet Union, where health care was characterised by a virtually exclusive role for the state in financing and delivery. There are common features to the reforms in three Baltic States. At the time of this project in 2002, a central ministry was responsible for overall health policy and a publicly-controlled insurance fund financed health care. Primary care was provided by family doctors reimbursed on a capitation basis, who were the gate-keepers for access to specialist services. In 2002, health services were still in transition with efforts at decentralisation, cost recovery and privatisation all in play to varying degrees.

Estonia is the smallest of the Baltic countries with a population of 1.3 million. It is also the most prosperous, with a per capita GNI at the time of the study (2002) of $4,140 (compared with Latvia $3,660 and Lithuania $3,480 in the same year, World Bank Atlas method, current US$). The Ministry of Social Affairs was responsible for health care. The Estonian Health Insurance Fund (EHIF), a public independent body, funded most primary, secondary and tertiary health care. EHIF paid for medical examinations and services, benefits in cases of temporary incapacity to work, part of the costs of prescribed medicines, and some health promotion activities [1-3]. Family doctors had the status of independent contractors [1,4] and almost all health services operated under contract with EHIF. In 2002 most hospitals were still owned by municipal governments, however, which ran them as limited companies or non-profit organizations. In addition to employed people, the EHIF also covered spouses of the insured, pensioners, children, disabled people, pregnant women and persons registered as unemployed [5]. Health care was officially free for all legal residents, except for a small consultation fee. Certain fees, such as those for visits to specialists, for home visits by family doctors, and hospital bed fees came into force since 2002.

Latvia lies between its two neighbours not only geographically bur also in terms of population (2.4 million) with a per capita GNI lower than Estonia's but roughly the same as that of Lithuania. At the time of the survey, the Latvia State Compulsory Health Insurance Agency (SCHIA) administered all government health care. Health care funds were controlled by SCHIA and disbursed through eight regional Sick Funds which contracted the various health care providers. Secondary and tertiary care were centralized and provided by national and municipal facilities, as well as private clinics. All Latvian citizens, permanent residents and those who have paid income tax for more than six months were entitled to a minimum basket of health care services which was revised every year [6,7]. All patients paid small consultation fees. Many procedures, however, required patient co-payments and some, such as dental care, joint replacement and infertility treatment were not covered at all.

Lithuania is the largest of the three countries in surface area and population (3.5 million). In 1994–95, Lithuania shifted the administration of health care services from the Ministry of Health to the ten counties. At the time of the social audit, the counties were in charge of the enforcement of the state health programs. Municipalities were responsible for primary health care (through family physicians) and for running small and medium size hospitals within their localities [8]. Financing for all public health care institutions, on the other hand, was centralised in the Statutory Health Insurance Fund (SHIF) which aimed to provide all permanent residents with all basic and essential health services free of charge. However the list of the specific services considered basic and essential was complex and often a source of confusion [8,9]. Users paid fully for certain services including dentistry, certification of health status, acupuncture, treatment of alcohol abuse, cosmetic procedures, therapeutic abortions and some nursing services. Numbers of private health care providers increased more rapidly in Lithuania than in the other Baltic countries [8,12].

Petty corruption in the health sector can manifest in several practices, including unofficial payments, salaries of "phantom" workers, use of official time for private consultations and privatisation of public supplies and services [10]. The exact mixture varies considerably from place to place, requiring local evidence to generate solutions for a particular context. Corruption in health care in countries of the former Soviet Union was recognised as a problem [10]. Studies on corruption in the health sector have been increasing [11,12], but one of the most recent reviews noted the surprisingly fragmented nature of the evidence, the lack of meaningful indicators for cross country comparison, and an absence of critical data at the country level [13].

Petty corruption – including the need to make unofficial payments to providers to obtain services [14] – is petty only in the criminal codes. Cumulatively, petty corruption can have a massive effect on services delivery. It increases the cost of key public services, limits access for those least able to pay [15,16] and inhibits the improvement of services and the ability of reforms to increase resources, raise services quality and responsiveness, increase efficiency, increase provider satisfaction, and achieve structural reforms [10,12]. In this way, petty corruption siphons off financial, technical and human resources that should maintain and improve the system. Some authors have argued that informal payments allow the system to function, but there are convincing arguments that the overall effects are negative at both individual and system levels [17]. Identifying and preventing corrupt practices should improve the functioning of the system and ultimately improve the well-being of people by building the capacity of government to meet its stated social and economic goals.

Partly because it deals with illegal activities, measurement of unofficial payments is not easy [18-20]. Interviewees are reluctant to speak about it. In Bulgaria, for example, around one third of a sample declined to answer these questions [21]. In Ghana, around one half of interviewees said they would not report an unofficial payment [22]. It is also difficult to distinguish between gifts as expressions of gratitude or as unofficial payments [23,24]. Another problem is that health service users might themselves not differentiate official from unofficial payments, as can be the case when public institutions are used for private health care. In Bangladesh, for example, unofficial payments almost invariable accompany official payments [25]. To avoid this problem, studies of unofficial payments in this setting have focused on conditions that should by law be free – in these cases, any payment is unofficial [26,27].

Attempts to measure unofficial payments have produced a wide range of results across a number of countries. With caveats about the comparability of data, Lewis presented data on unofficial payments from 12 countries in Eastern Europe and Central Asia – a range between 21% and 91% [23]. Studies in Tajikistan [28], Russia [29], Bulgaria [21], Poland [30], Albania [31], Slovakia [32], Hungary [17], Cambodia [33], South Korea [34], South Africa [35] and Cameroon [36] reported a range of unofficial payment rates to health workers from 1% to 85%. The problem about these studies is that they did not use a standard methodology – making comparison all but meaningless.

In 2002, we worked with national counterparts in the Baltic States to examine unofficial payments in the health sector. Our regional social audit had four inter-dependent objectives: to assemble evidence on the incidence of unofficial payments in the last contact with health services; to measure the public perception of corruption; to document links between perception, attitudes and concrete experience; and to suggest actionable steps to improve the situation. This paper reports on the findings of the surveys in the three countries regarding unofficial payments in health services, from the perspective of the public and service users.

## Methods

A series of interviews with planners and decision makers set the limits of the enquiry. The government counterparts in each country identified the components of health service delivery, especially those related to informal payments, which would benefit from community-based information. We used the social audit methods developed by CIET over the last two decades [35,37,38] which collect information about public services from beneficiaries and service providers, using this as a basis for involving stakeholders in discussing the findings and making changes to improve the services. The methods have been used to measure impact, coverage and cost in the fields of environment [39], urban transport [40], agriculture [41] and judiciary [42], and for providing evidence for community-designed strategies to combat corruption in public services [43].

### Sample and sampling

National statistical departments in each country supported the sampling process by providing the sampling frame of enumeration areas based on the most recent census. In each country we drew a random cluster sample of 30 enumeration areas, stratified by region and by rural/urban/capital location. Within each enumeration area the site comprised some 110 contiguous households, radiating out from a randomly selected starting point, with no sub-sampling within the site. This sampling strategy allows community level variables as well as household and individual variables to be included in analysis of outcomes of interest [27,38,44]. The final sample across all three countries was 10,320 households representing some 25,000 people (Table 1).

**Table 1:** The sample for the Baltics social audit

	**Households interviewed**	**People represented**	**Health institutions reviewed**	**Community focus groups**	**Health worker focus groups**
**Estonia**	3,388	7,526	33	30	1
**Latvia**	3,439	8,926	41	30	3
**Lithuania**	3,493	8,541	30	30	2

**TOTAL**	10,320	24,993	104	90	6

### Data collection

In May and June 2002 trained local interviewers conducted a household survey in the sample sites to collect information on household views and individual client experiences with the health sector. Except for a few country-specific questions, we used the same survey instrument across all three countries. After translation into Estonian, Latvian, Lithuanian, and Russian, independent language consultants back translated it to check preservation of meaning. The instrument asked households whether they considered unofficial payments to health care professionals as corruption, how they rated corruption in government health services (on a five point scale from very low to very high), and whether the level of corruption in health services had decreased, stayed the same, or increased in the last three years. We used respondent-estimated household income to illustrate the likely economic impact on the population of health care costs, including gifts and unofficial payments. The interviewer asked: "Adding up the incomes of all the household members, what is the average monthly income of this household?"

Adult members of households gave information about their last contact with health services during the last five months (this coincided with the beginning of the calendar year), as a period for which they were likely to have reasonable recall. They answered questions about the type of contact, the type of health service, and their time on any waiting list. Questions about official and unofficial payments and gifts (separately) included timing, amounts, recipients, and perceived effects.

Trained facilitators returned to each sample community and conducted a focus group discussion with community members. They present key findings from the household survey to the participants and to invite their views and suggestions for improvements. We also conducted focus groups of doctors and nurses (two groups in Lithuania, three groups in Latvia and one group in Estonia) to explore their views about the findings and to seek their suggestions for corrective strategies. In each focus group a reporter took notes during the discussion and the facilitator and reporter together prepared a report on each focus group. We translated the focus group reports into English and a small group of researchers in each country (including nationals of that country) went through the reports and identified emerging themes.

### Analysis

We calculated frequencies of key outcomes for each country, including household opinions of the government health services, their rating of corruption in these services, and government service users' reports of making unofficial payments or giving gifts. We investigated variables related to these outcomes using multiple logistic regression analysis (step-down from a saturated model) to identify possible risk factors for unofficial payments. The saturated model included all variables related to the outcomes in univariate analyses: age, sex and education of respondents, urban or rural residence, language spoken (national language or not), whether households considered their income was sufficient for their needs, and whether respondents had enough information about their entitlements.

To facilitate their use in the analysis, we dichotomised some of the independent variables. Prior experience and the stakeholder design processes guided this dichotomisation. We divided age of respondent and of the breadwinner into those below and above 50 years of age; and education of the respondent and breadwinner into "basic" (the legal minimum education) and higher.

We used CIETmap [45] for the analysis and population-weighted raster maps. Contrasts are reported as the odds ratio (OR), with 95% confidence intervals (CI). The OR figures shown are the adjusted values from the logistic regression models. The tables in the main paper showing OR figures are each derived from the results of several different logistic regression analyses in each of the three countries. Details of the logistic regression models are included in the supplementary data file [see Additional File 1].

### Maps and their interpretation

Key findings from community-based questionnaires are represented in population-weighted raster maps [45]. The interpretation of these maps is straightforward, not unlike a weather map. Darker colours on the map represent higher levels of the indicator being mapped, as if the population represented by each selected site were 'spread out' on the geographic surface. The positions of colour changes between sample sites are determined by a process of interpolation, taking into account the relative populations of sites. Population weighting thus transforms the geographic space into population space. For example, if 30% of the map falls into a given range of the indicator, then 30% of the population of the country falls within that range. As with a standard weather map, trends are much more accurate than the exact location of any contour gradient. In interpreting the maps, one should not be looking at the colour at a specific point, but at the colour change trends.

### Communication of Results

In each of the three countries, stakeholders, including health officials, health providers and representatives of civil society, participated in workshops to discuss the survey results and identify items that could be acted on to make positive changes to the system. A communication strategy in each country proposed main actionable findings.

### Ethical review

The CIETinternational Ethical Review Board reviewed and approved the project in May 2002.

## Results

### Perceptions of unofficial payments

Only about half the households thought an unofficial payment to a health care professional was a form of corruption, with little difference between the three countries (Table 2). More than a third of households across the region (Estonia 44%, Latvia 38%, and Lithuania 34%) said they would be willing to report health care professionals who demanded unofficial payments. A high proportion declined to respond to this question or said they did not know what they would do (Estonia 15.5%, Latvia 16.3% and Lithuania 7.2%.)

**Table 2:** Proportion of households that consider unofficial payments to be corruption

	**Number of households responding**	**Number (%) that considers unofficial payments to be corruption**
**Estonia**	2851	1588 (56)
**Latvia**	3015	1529 (51)
**Lithuania**	3205	1774 (55)

The community focus groups reflected this ambivalence about whether unofficial payments to health care workers were a form of corruption. An exchange in a group in Estonia illustrates this: "It's not corruption if you pay afterwards" to which another person replied: "You pay well and the next time they are waiting for you already. It is corruption." Some thought it was not corruption unless the payment was requested "If I pay my own money and no one is demanding it from me, I don't think this is corruption." In the focus groups of doctors and nurses there was also disagreement about whether unofficial payments were corruption, with some participants suggesting that payments in cash or kind after treatment represented gratitude rather than corruption, and others pointing to the long tradition of paying health workers.

When households were asked their opinions about corruption in the government health services, a significant minority in each country felt unable to give a rating (Estonia 36%, Latvia 28%, Lithuania 12%). Of those that gave an opinion, more people in Lithuania thought the level of corruption was high or very high (Table 3). In Estonia and Latvia, respondents speaking the national language were less likely to rate corruption as high (Table 4) [see Additional File 1]. Similarly, many people were unable to say if corruption in the health services had changed in the last three years (Estonia 42%, Latvia 39%, Lithuania 23%), but of those who gave an opinion, more people in Lithuania considered it had increased (Estonia 49% (846/1715), Latvia 50% (1032/2053), Lithuania 57% (1527/2659).

**Table 3:** Proportion of households that consider the level of corruption in the health services to be high

	**Number of households responding**	**Number (%) that considers level of corruption to be high**
**Estonia**	2004	863 (43)
**Latvia**	2446	1092 (45)
**Lithuania**	3045	1957 (64)

**Table 4:** Variables related to household perception of corruption in government health services as high

**Related variable**	**Adjusted Odds Ratios (95% CI)**		
	*Estonia*	*Latvia*	*Lithuania*

Speaking national language	OR 0.78 (0.63–0.96)	OR 0.74 (0.62–0.89)	
Having enough knowledge about entitlements	OR 0.73 (0.61–0.89)		OR 0.75 (0.63–0.90)

The OR and 95% CI are the adjusted values from logistic regression analysis. See Methods for the variables included in the analysis and details of the models from individual countries in the supplementary tables

### Prevalence of unofficial payments and gifts

**Figure 1:**
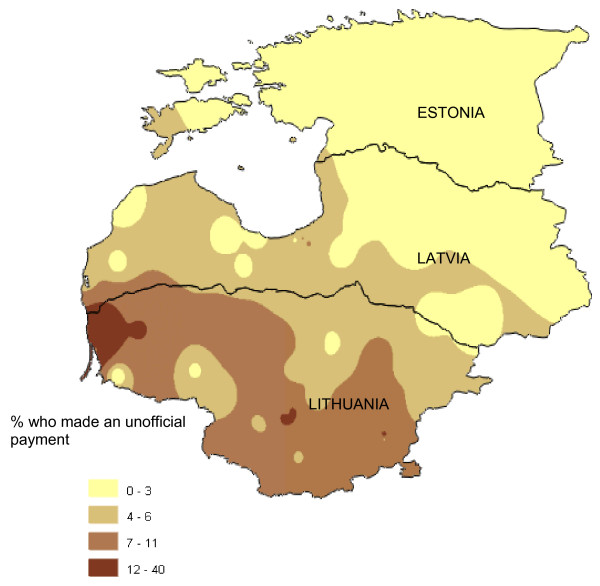
**Geographical distribution of unofficial payments made during most recent contact with government health services**. This is a population weighted raster map of percent of households who made an unofficial payment in last contact with government health services, showing trends for the variable rather than absolute values at any given point on the map.

Despite these attitudes and perceptions, very few household members who had used government health services in the first five months of 2002 said they had made an unofficial payment in their last contact (Table 5). The reported number of unofficial payments was lowest in Estonia (0.7%) and highest in Lithuania (8%). Figure 1 shows the geographical variation in the proportion of service users making unofficial payments. The highest rates were in the south and west of Lithuania. In all three countries, unofficial payments were more frequently reported in contacts with specialist doctors, and half or more of the payments were made before or during the treatment (Table 5). Declining to answer the question was uneven across three countries (Estonia 12.3%, Latvia 1.7%, and Lithuania 8.1%).

**Table 5:** Service users who gave unofficial payments and gifts in their last contact with government health services: type of care and timing of payments

	**Unofficial payments**			**Gifts**		
	**Number of service users responding**	**Number (%) making unofficial payments**		**Number of service users responding**	**Number (%) giving gifts**	

		*Overall:*			*Overall:*	

**Estonia**	2456	18 (0.7)		2475	309 (13)	
**Latvia**	3177	96 (3)		3185	436 (14)	
**Lithuania**	2553	211 (8)		2588	349 (14)	

		*By type of care:*			*By type of care:*	

		General care	Specialist care		General care	Specialist care

**Estonia**	2435	3/1338 (0.2)	14/1097 (1.3)	2454	146/1360 (11)	161/1094 (15)
**Latvia**	3143	39/2004 (2)	54/1139 (5)	3150	220/1999 (11)	212/1151 (18)
**Lithuania**	2511	66/1229 (5)	140/1282 (11)	2548	151/1249 (12)	193/1299 (15)

		*By timing during treatment:*			*By timing during treatment:*	

		Before/during	After		Before/during	After

**Estonia**		8/16 (50)	8/16 (50)		49/292 (17)	243/292 (83)
**Latvia**		42/89 (47)	47/89 (53)		128/423 (30)	295/423 (70)
**Lithuania**		133/198 (67)	65/198 (33)		106/335 (32)	229/335 (68)

The community and health worker focus groups discussed the low rate of reported unofficial payments from the household survey. Many community focus group participants thought the actual rate of making unofficial payments for health care was higher: "You cannot get any attention in a hospital these days if you don't bribe someone there" (Lithuania). They suggested that people may be unwilling to admit to making unofficial payments: "Who wants to confess?" (Estonia); "People did not want to admit they paid because they got something back as a benefit from payment." (Latvia)

By contrast, in Lithuania, the doctors and nurses suggested the 8% rate of unofficial payments was an over-estimate: "People don't have enough money to pay – there is just a lot of talk about it." and "If gifts are included, these numbers are about right." But in Latvia, health workers thought the rates of unofficial payments from the household survey were an underestimate: "The actual number who pay is higher, but mainly for expensive specialists." In Estonia, doctors considered the low reported rate of unofficial payments may reflect reality: "Only very desperate people are doing this."

**Figure 2:**
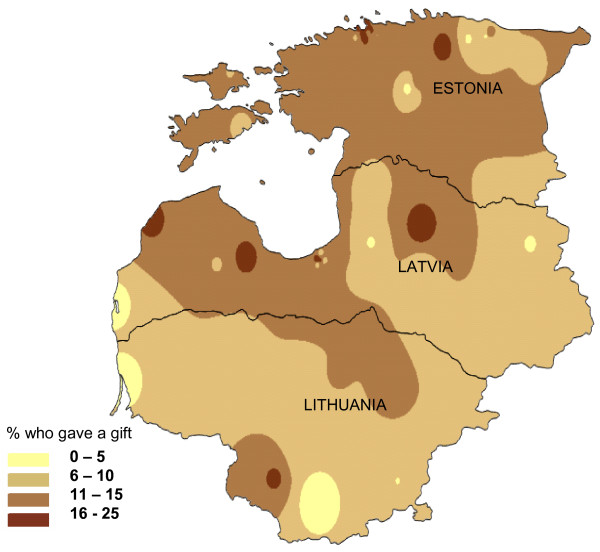
**Geographical distribution of gifts given during most recent contact with government health services**. This is a population weighted raster map of percent of households who gave a gift in last contact with government health services, showing trends for the variable rather than absolute values at any given point on the map.

Giving gifts was more common, and in this case did not vary much between the three countries, with around 14% of respondents in each country reporting they gave a gift in their last contact with government health services. Gifts were more common in specialist care than in general care, and most of the gifts were given after treatment (Table 5). Figure 2 shows the geographical spread of giving gifts.

In all three countries, those who gave gifts were at least three times more likely to have also made an unofficial payment. In Latvia and Lithuania, respondents with more education were more than twice as likely to report making an unofficial payment (Table 6) [See Additional File 1].

**Table 6:** Variables related to unofficial payments in government health services

**Related variable**	**Adjusted Odds Ratios (95% CI)**		
	*Estonia*	*Latvia*	*Lithuania*

Giving a gift	OR 11.34 (5.07–25.35)	OR 4.04 (2.72–6.00)	OR 3.40 (2.52–4.49)
More education		OR 2.53 (1.69–3.80)	OR 2.10 (1.55–2.86)
Urban residence		OR 2.41 (1.45–5.26)	
Household income sufficient for needs			OR 1.53 (1.04–2.14)

The OR and 95% CI are the adjusted values from logistic regression analysis. See Methods for the variables included in the analysis and details of the models from individual countries in the supplementary tables

### Relative value of unofficial payments and gifts

Table 7 presents the unofficial payments made by those who said they made such payments during their most recent encounter with government health services. For comparison purposes we also record estimated monthly household income among all those who made either unofficial payments or gifts. In Latvia the median unofficial payment was 6% and in Lithuania 9% of median declared monthly income.

**Table 7:** Value of unofficial payments and gifts compared with monthly household income, in Euros

	**Payments by individuals, last contact w. system**		**Gifts by individuals, last contact with the system**		**Monthly household income among those making unofficial payments or gifts**	
	**Mean**	**Median**	**Mean**	**Median**	**Mean**	**Median**

**Estonia**	116	13	7	3	339	256
**Latvia**	45	17	7	3	393	284
**Lithuania**	46	20	10	6	320	231

The vast majority of gifts (Estonia 98%, Latvia 95%, Lithuania 93%) were in kind (candy, flowers, liquor, etc) rather than in money. Respondents who gave gifts were asked to estimate the value of the gift. The Euro equivalent of these estimates, also presented in Table 7, represents from 1% to 4% of monthly household income.

We asked all respondents what they considered the maximum acceptable value of a gift to a health care professional. Up to one third in each country would not agree to any size gift (Estonia 31%, Latvia 31%, and Lithuania 23%). Among those who considered gift giving permissible, acceptable values mentioned were similar to those of the actual gifts given.

### Benefits derived from unofficial payments

People who made unofficial payments in government health services answered a separate question about the benefit they received from making the payment. Some reported positive results such as quicker service, better quality of service and a personal sense of satisfaction or expressed gratitude from the health professional. However, a sizeable minority of those who made an unofficial payment said they did not receive any benefit from doing so (Estonia, 1/12; Latvia, 26/77; Lithuania, 49/177).

### Willingness to pay

Table 8 shows the percentages of those households who said they were willing to pay for specific changes (improvements) in family doctor services and specialist services they would like to see, and the maximum amounts they would be willing to pay for these improvements. Among those who were willing to pay, the median amounts ranged from 1% to 3% of median declared monthly household income. A large proportion did not answer this question (Estonia 16.1%, Latvia 41.7% and Lituania 14.7%).

**Table 8:** Willingness to pay for improvements in services of family doctors and specialists

	**Family doctor**			**Specialist services**		
	**Proportion willing to pay**	**Maximum amount willing to pay (in Euros)**		**Proportion willing to pay**	**Maximum amount willing to pay (in Euros)**	
				
		**Mean**	**Median**		**Mean**	**Median**

**Estonia**	27%	7	2	40%	5	3
**Latvia**	55%	4	2	71%	8	3
**Lithuania**	41%	7	6	35%	9	6

The amounts shown are the mean and median only among those willing to pay anything

**Figure 3:**
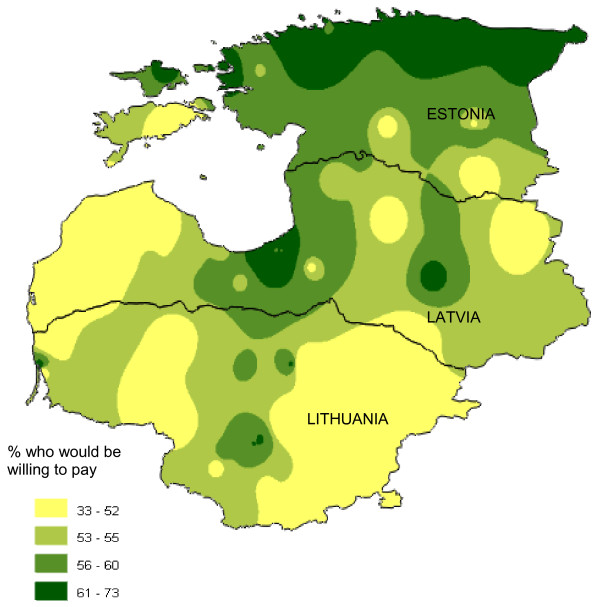
**Geographical distribution of willingness to pay to avoid waiting lists for surgery or other hospital treatment**. This is a population weighted raster map of percent of households who would be willing to pay to avoid waiting lists for surgery or other hospital treatment, showing trends for the variable rather than absolute values at any given point on the map.

In addition, households were specifically asked if they would be willing to pay to avoid waiting lists for surgery or other hospital treatment. More than one half of households said they would be willing to pay for this, especially in Estonia (Estonia 62%, 1918/3100; Latvia 56%, 1730/3086; Lithuania 51%, 1695/3309). Figure 3 shows how willingness to pay to avoid waiting lists for surgery is distributed geographically. The greatest willingness to pay appeared in the more prosperous northern area of Estonia and in Riga, the capital of Latvia. In the relatively poorer Lithuania, people were least willing to pay. In all three countries, respondents under the age of 50 years were three or four times more likely than older people to be willing to pay to avoid waiting lists, and households reporting enough income for their needs were more likely to be willing to pay (Table 9) [See Additional File 1].

**Table 9:** Variables related to willingness to pay to avoid a waiting list

**Related variable**	**Adjusted Odds Ratios (95% CI)**		
	*Estonia*	*Latvia*	*Lithuania*

Age less than 50 years	OR 4.75 (4.03–5.59)	OR 4.73 (4.04–5.55)	OR 3.26 (2.78–3.82)
Household income sufficient for needs	OR 1.45 (1.22–1.72)	OR 1.94 (1.58–2.37)	OR 1.52 (1.26–1.85)

The OR and 95% CI are the adjusted values from logistic regression analysis. See Methods for the variables included in the analysis and details of the models from individual countries in the supplementary tables

Community focus groups discussed the findings about paying to avoid waiting lists and whether it was fair or right for people to do this. Some participants thought it was fine to jump the queue if you could afford to pay: "I can pay, and I am going before others; let the others wait." (Latvia). Others thought it was unfair for those who could not afford to pay: "If I have money, I get served first, and if I am poor, I can wait forever?" (Lithuania) or that it would only be acceptable if there were two separate systems or if people who could pay went privately "People who can pay may do this but the doctors should deal with them after official working hours" (Estonia). Some mentioned that such payments should not be necessary as people paid taxes for their health care: "If we did not have to pay taxes, it would be okay to pay extra." (Lithuania)

Focus groups of health workers often expressed that they were not in favour of people paying to avoid waiting lists, but recognised it was happening. Some thought the possibility to pay could be made official: "Anyway, people are paying to skip waiting: it has to be made official" (Latvia). Others thought the practice should be stopped: "It is unfair to those who cannot pay. It should be stopped and people who allow it to happen must be punished." (Lithuania), and others suggested there should be a separate system for those paying: "It has to be working out of official hours" (Estonia)

## Discussion

### Study limitations

This study has the well known limitations of a cross-sectional study. It provides a snapshot of the situation at one time point during a period of adaptation to post-Soviet conditions. All household information is based on self-reporting and some respondents may have been reluctant to admit making unofficial payments. Focus group participants in Latvia and Lithuania felt that unofficial payments were under-reported, mainly because people did not want to admit to making these payments. Another reason for under-reporting unofficial payments is that people may not be aware of which payments are official and which are not.

Several authors point to the difficulties of definition and of under-reporting [15,18-20]. A comparative study, based on surveys in eight former Soviet Union countries in 2001 reported a range of out-of-pocket payments (including money and/or gifts) from 65% and 56% in Georgia and Armenia, to 8% and 19% in Belarus and Russia [46]. Our figures for unofficial payments and gifts combined are comparable with those at the lower end of this range.

Although we anticipate under-reporting of unofficial payments, the relative frequency among the three countries is probably still interpretable. Perceptions of corruption in the health sector, though much higher than actual reporting of informal payments, showed a similar pattern across the three countries: lowest in Estonia and highest in Lithuania with Latvia in the middle.

Another problem comes in relating unofficial payments to health costs and household income. While reliable estimates of *absolute *household income require much more complex questioning and documentation [47,48], our concern was to obtain a measure against which to assess the *relative *size of health costs, unofficial payments and willingness to pay.

### Are unofficial payments corruption?

Nearly one half the respondents to our survey did not consider unofficial payments to be corruption. In a separate Lithuanian study [8], only payments in cash were perceived as bribes. In the household interviews we asked separately about unofficial payments and gifts, and focus groups distinguished cash payments and gifts. Among cash payments both the size of the payment and whether it was given before or after the service helped to determine whether it should be considered a bribe or not.

In this cross-sectional study we cannot be sure of the direction of causality; it could be that giving gifts and making unofficial payments are both manifestations of some underlying process. However, the very similar rate of gifting (13–14%) in the three countries contrasts strikingly with the very different rates of unofficial payments across the countries (0.7% in Estonia, 3% in Latvia and 8% in Lithuania). This raises the question whether gifting influences unofficial payments (rather than vice versa) in a way that is mediated through the particular health care reform approaches in the three countries.

Most of the gifts reported in our survey were made in kind, 93% in Lithuania to 98% in Estonia, and were generally given after treatment. We attempted to estimate the value of the gifts; in some cases (such as when the gift was simply a bunch of flowers) this was a somewhat artificial exercise. The median of the estimated value of gifts (including all types) was a third or less the median value of reported unofficial payments. In community focus groups in all three countries, people overwhelmingly supported the practice of giving gifts to health care professionals, stating that gifts were an expression of gratitude for care. There is evidence from other sources of a culture of gift giving in the context of professional services in regions of the former Soviet Union [12]. In Estonia, where less than 1% of respondents reported making unofficial payments, the level of gift-giving was similar to that of the other two countries (Table 5 and Figure 2).

But less benign interpretations are possible. Under the Soviet system, the chief mechanism for rationing health care was making people wait. Gifts in kind help to establishing a personal relationship with the caregiver and they easily lead to favouritism as far as waiting times are concerned [12]. It may be noteworthy that those who gave gifts were significantly more likely to have also made an unofficial payment. And there appear to be substantial minorities in each country that consider it inappropriate to give gifts of any value at all.

The lack of consensus on whether informal payment is corruption is a subject for concern. Some believe that the very lack of consensus encourages corruption [49].

### Perceptions *vs*. actual experience of corruption

In our study, perception of corruption is higher than actual experiences of unofficial payments in health services. This contrast could be explained in part by under-reporting of unofficial payments actually made as well as by the fact that the perceptions are based on a lifetime of experience, information from friends and neighbours and media reports. But it may also mean that relatively low levels of corruption in health services have powerful ripple effects in the public opinion. People may also be including forms of corruption other than petty corruption when they rate the overall level of corruption in the health services.

### Willingness to pay and the formalisation of informal payments

The current discussion as to whether formalisation of informal payments might lead to a reduction in corruption [12,50] could be better informed by data concerning health care users' actual willingness to pay for better service. Our survey indicates that in 2002 willingness to pay was closely related to the ability to pay and that the amounts people said they were willing to pay were not very large (Tables 8 and 9 and Figure 3). Overall, the margin of acceptance for across-the-board increases in health service charges, even when accompanied by improvements, appeared to be quite narrow, and even taking into account the willingness of many to reward individual acts of good service with gifts.

## Conclusion

Estonia, with the simplest health reform structures, evidenced the least corruption at both the level of experience and that of perceptions. Latvia, with higher official user costs, was in the middle. Lithuania, larger and more complex in its approach to health reform, evidenced the highest levels of unofficial payments, and the greatest mistrust towards the system. As each country pursues its own approach to health care reform, the information from this 2002 study can serve as a baseline for examining how effective the different reforms have been in the effort to control petty corruption.

## Competing interests

The authors declare that they have no competing interests.

## Authors' contributions

AC oversaw fieldwork, assisted with data analysis, drafting of the country reports, workshop presentations, and drafting of this paper. SPS coordinated the field work in the three countries, managed the project in Latvia and assisted with data analysis. NA was the principal investigator with overall responsibility for project design and implementation as well as for analysis of the results and drafting of this paper. MR assisted with project management in Estonia and Latvia and compilation of a three-country report. DC and DM managed the project in Estonia and assisted with data analysis. SMe managed the project in Lithuania and assisted with data analysis. EK assisted with the project in Lithuania and in the subsequent analysis and reporting across all three countries. RJL assisted with analysis of the economic data and drafting of this paper. SMi developed the maps and contributed to the analysis. All authors read and approved the final manuscript.

## Pre-publication history

The pre-publication history for this paper can be accessed here:



## Supplementary Material

Additional file 1 Findings from individual country logistic regression analyses. The file shows the final models of logistic regression analyses for each country separately. Tables 4, 6 and 9 in the report are based on these models. The numbering of the tables in the file corresponds with the numbering of the relevant tables in the main text. Click here for file

## References

[B1] Meiesaar K, Lember M (2004). Efficiency and sustainability of using resources in Estonian primary health care. Croatian Medical Journal.

[B2] Jesse M, Habicht J, Asviksoo A, Koppel A, Irs A, Thomson S (2004). Health care in systems in transition: Estonia.

[B3] Polluste K, Mannik G, Axelsson R (2005). Public health reforms in Estonia: impact on the health of the population. BMJ.

[B4] Lember M (2002). A policy of introducing a new contract and funding system of general practice in Estonia. International Journal of Health Planning and Management.

[B5] Estonian Health Insurance Fund. http://www.haigkassa.ee/eng/ehif/.

[B6] Brown H (2004). Looking to the future in Latvia. The Lancet.

[B7] Karashkevica J (2004). Latvia's health care system on the move. Cahiers de Sociologie et de Demographie Medicales.

[B8] Jakusovaite I, Darulis Z, Zekas R (2005). Lithuanian health care in transition state: ethical problems. BMC Public Health.

[B9] Dobrevolskas A, Buividas R (2003). Study of the Social Protection Systems of the 13 Applicant Countries: Lithuania.

[B10] Thompson R, Witter S (2000). Informal payments in transitional economies: implications for health sector reform. International Journal of Health Planning and Management.

[B11] Transparency International Global Corruption Report 2006: Special Focus – Corruption in Health.

[B12] Ensor T (2004). Informal payments for health care in transition economies. Social Science & Medicine.

[B13] Lewis M (2006). Governance and corruption in public health care systems. Center for Global Development.

[B14] Sturges P (2004). Corrruption, Transparency and a Role for ICT?. International Journal of Information Ethics.

[B15] Falkingham J (2004). Poverty, out-of-pocket payments and access to health care: evidence from Tajikistan. Social Science and Medicine.

[B16] Szende A, Culyer AJ (2006). The inequity of informal payments for health care: the case of Hungary. Health Policy.

[B17] Gaal P, McKee M (2005). Fee-for-service or donation? Hungarian perspectives on informal payment for health care. Social Science and Medicine.

[B18] Gaal P, Belli PC, McKee M, Szocska M (2006). Informal Payments for Health Care: Definitions, Distinctions, and Dilemmas. Journal of Health Politics Policy and Law.

[B19] Ensor T, Savelyeva L (1998). Informal payments for health care in the former Soviet Union: some evidence from Kazakstan. Health Policy and Planning.

[B20] Miller WL, Groenland AB, Koshenhkina TY (2000). 'If you pay, we'll operate immediately'. Journal of Medical Ethics.

[B21] Delcheva E, Balabanova D, McKee M (1997). Under-the-counter payments for health care: evidence from Bulgaria. Health Policy.

[B22] Ofori-Atta AL, Gadzekpo A The cost of corruption in health institutions. 9th International Anti-corruption Conference.

[B23] Lewis M (2000). Who is paying for health care in Eastern Europe and Central Asia?. World Bank Europe and Central Asia Region.

[B24] Balabanova D, McKee M (2002). Understanding informal payments for health care: the example of Bulgaria. Health Policy.

[B25] Killingsworth RJ, Hossain N, Hedrick-Wong Y, Thomas S, Rahman A, Begum T (1999). Unofficial fees in Bangladesh: price, equity and institutional issues. Health Policy and Planning.

[B26] Thompson R, Xavier A (2002). Unofficial payments for acute state hospital care in Kazakhstan. A model of physician behaviour with price discrimination and vertical service differentiation.

[B27] Cockcroft A, Andersson N, Milne D, Hossain MD, Karim E (2007). What did the public think of health services reform in Bangladesh? Three national community-based surveys 1999–2003. Health Research Policy and Systems.

[B28] Falkingham J (2004). Poverty, out-of-pocket payments and access to health care: evidence from Tajikistan. Social Science & Medicine.

[B29] Feeley FG, Sheiman IM, Shishkin SV (1999). Health sector informal payments in Russia.

[B30] World Bank Experience with corruption in the health sector in Poland. http://www.worldbank.org/publicsector/anticorrup/IACC/HealthPoland.doc.

[B31] Vian T, Gryboski K, Sinoimeri Z, Hall CR (2004). Informal payments in the public health sector in Albania: A qualitative study.

[B32] World Bank Corruption in Slovakia (2000). Results of Diagnostic Surveys, Prepared at the request of the Government of the Slovak Republic.

[B33] Barber S, Bonnet F, Bekedam H (2004). Formalizing under-the-table payments to control out-of-pocket hospital expenditures in Cambodia. Health Policy and Planning.

[B34] Mc Manus J (1999). South Korea cracks down on medical corruption. BMJ.

[B35] Andersson N, Matthis J, Paredes S, Ngxowa N (2004). Social audit of provincial health services: Building the community voice into planning in South Africa. Journal of Interprofessional Care.

[B36] Van der Geest S (1982). The efficiency of inefficiency: Medicine distribution in south Cameroon. Social Science and Medicine.

[B37] Andersson N, Martinez E, Cerrato F, Morales E, Ledogar R (1989). The use of community-based data in health planning in Mexico and Central America. Health Policy and Planning.

[B38] Andersson N, Roche M (2006). Gender and evidence-based planning: the CIET methods. Development in Practice.

[B39] Arostegui J, Andersson N (1995). Nicaragua: Impact of the National Environmental Program.

[B40] Arostegui J, Andersson N (1995). Results-oriented management of Managua urban public transport.

[B41] Cockcroft A (1996). Performance and perceptions of health and agriculture services in Uganda.

[B42] Massoud N (1995). Measuring client satisfaction and expectations: The Case of the Mali Public Service.

[B43] Cockcroft A, Legorreta J (1998). National Integrity Survey, Uganda.

[B44] Andersson N, Palha de Sousa C, Paredes S (1995). Social cost of land mines in four countries: Afghanistan, Bosnia, Cambodia and Mozambique. British Medical Journal.

[B45] Andersson N, Mitchell S (2006). Epidemiological geomatics in evaluation of mine risk education in Afghanistan: introducing population weighted raster maps. Int J Hlth Geographics.

[B46] Balabanova D, McKee M, Pomerleau J, Rose R, Haerpfer C (2004). Health service utilization in the former Soviet Union: evidence from eight countries. Health Services Research.

[B47] US Department of Health and Human Services, Assistant Secretary for Planning and Evaluation (2003). "Leavers" and Diversion Studies: Household Income.

[B48] Isaacs J Total Household Income: Overview of Measures Used in ASPE Welfare Outcomes Grantee Surveys with Welfare Leavers [Prepared for: Fall 1999 ASPE Welfare Outcomes grantee Meeting].

[B49] Open Society Institute (2002). Corruption and anti-corruption policy in Latvia. Monitoring the EU accession process: Corruption and anti-corruption policy.

[B50] Allin S, Davaki K, Mossialos E Paying for 'free' health care: The conundrum of informal payments in post-communist Europe. Transparency International Global Corruption Report 2006: Special Focus – Corruption in Health.

