# Supplementary material for: An inter-country comparison of unofficial payments: results of a health sector social audit in the Baltic States
Source: BMC Health Serv Res. 2008 Jan 21;8:15. doi: 10.1186/1472-6963-8-15 (PMC2233619; doi:10.1186/1472-6963-8-15)
Supplement: Additional file 1 — Findings from individual country logistic regression analyses. The file shows the final models of logistic regression analyses for each country separately. Tables 4, 6 and 9 in the report are based on these models. The numbering of the tables in the file corresponds with the numbering of the relevant tables in the main text. [file 1472-6963-8-15-S1.doc]

**Findings from individual country logistic regression analyses**

Table 4. Variables related to household perception of level of corruption in government health services.

Logistic regression models on which table 4 in the main text is based:

Estonia

+-----------+--------+------+-------------+-------------+-------------+----------+-------------+----------+

| Q34RATE1 | crude | wt | 95% CI | 90% CI | 99% CI | MH Summ | test for | p-value |

| | OR | OR | wt OR | wt OR | wt OR | Chi Sq | interaction | |

+-----------+--------+------+-------------+-------------+-------------+----------+-------------+----------+

| Q4LANG1 | 0.74 | 0.78 | 0.63 - 0.96 | 0.65 - 0.93 | 0.59 - 1.03 | 5.29 | 3.0046 | 0.0830 |

+-----------+--------+------+-------------+-------------+-------------+----------+-------------+----------+

| Q20KNOW1 | 0.71 | 0.73 | 0.61 - 0.89 | 0.63 - 0.86 | 0.57 - 0.94 | 10.12 | 3.0064 | 0.0829 |

+-----------+--------+------+-------------+-------------+-------------+----------+-------------+----------+

Latvia

+-----------+--------+------+-------------+-------------+-------------+----------+-------------+----------+

| Q34RATE2 | crude | wt | 95% CI | 90% CI | 99% CI | MH Summ | test for | p-value |

| | OR | OR | wt OR | wt OR | wt OR | Chi Sq | interaction | |

+-----------+--------+------+-------------+-------------+-------------+----------+-------------+----------+

| Q79SUFF1 | 0.71 | 0.71 | 0.58 - 0.87 | 0.60 - 0.84 | 0.54 - 0.92 | 11.33 | 2.4320 | 0.4877 |

+-----------+--------+------+-------------+-------------+-------------+----------+-------------+----------+

| Q4LANG1 | 0.72 | 0.74 | 0.62 - 0.89 | 0.64 - 0.86 | 0.58 - 0.94 | 10.26 | 3.2083 | 0.3606 |

+-----------+--------+------+-------------+-------------+-------------+----------+-------------+----------+

| Q3WINEDU1 | 1.38 | 1.40 | 1.19 - 1.65 | 1.22 - 1.61 | 1.13 - 1.74 | 16.06 | 1.9250 | 0.5881 |

+-----------+--------+------+-------------+-------------+-------------+----------+-------------+----------+

Lithuania

+------------+--------+------+-------------+-------------+-------------+----------+-------------+----------+

| Q34RATE1 | crude | wt | 95% CI | 90% CI | 99% CI | MH Summ | test for | p-value |

| | OR | OR | wt OR | wt OR | wt OR | Chi Sq | interaction | |

+------------+--------+------+-------------+-------------+-------------+----------+-------------+----------+

| Q2RESPSEX1 | 0.82 | 0.82 | 0.69 - 0.98 | 0.71 - 0.95 | 0.66 - 1.03 | 4.89 | 13.8635 | 0.0537 |

+------------+--------+------+-------------+-------------+-------------+----------+-------------+----------+

| Q79SUFF1 | 0.67 | 0.69 | 0.57 - 0.83 | 0.58 - 0.81 | 0.53 - 0.89 | 14.41 | 15.0928 | 0.0348 |

+------------+--------+------+-------------+-------------+-------------+----------+-------------+----------+

| Q3WINEDU1 | 0.67 | 0.67 | 0.53 - 0.85 | 0.55 - 0.82 | 0.49 - 0.92 | 10.67 | 15.8164 | 0.0268 |

+------------+--------+------+-------------+-------------+-------------+----------+-------------+----------+

| Q20KNOW1 | 0.75 | 0.75 | 0.63 - 0.90 | 0.64 - 0.88 | 0.59 - 0.96 | 9.24 | 8.7661 | 0.2699 |

+------------+--------+------+-------------+-------------+-------------+----------+-------------+----------+

Table 6. Variables related to making unofficial payments in government health services

Logistic regression models on which table 6 in the main text is based:

Estonia

+------------+--------+-------+--------------+--------------+--------------+----------+-------------+----------+

| Q65UNOFF1 | crude | wt | 95% CI | 90% CI | 99% CI | MH Summ | test for | p-value |

| | OR | OR | wt OR | wt OR | wt OR | Chi Sq | interaction | |

+------------+--------+-------+--------------+--------------+--------------+----------+-------------+----------+

| Q73GIFT1 | 13.69 | 11.34 | 5.07 - 25.35 | 5.77 - 22.28 | 3.94 - 32.65 | 34.99 | 1.8213 | 0.6103 |

+------------+--------+-------+--------------+--------------+--------------+----------+-------------+----------+

| Q36AGE50 | 2.43 | 3.30 | 1.17 - 9.33 | 1.38 - 7.90 | 0.84 - 12.93 | 5.09 | 1.7279 | 0.6308 |

+------------+--------+-------+--------------+--------------+--------------+----------+-------------+----------+

| Q4LANG1 | 0.27 | 0.38 | 0.14 - 0.99 | 0.17 - 0.85 | 0.11 - 1.34 | 3.94 | 0.9586 | 0.8113 |

+------------+--------+-------+--------------+--------------+--------------+----------+-------------+----------+

Latvia

+-----------------------+--------------+----------+------------------------+-----------------------+------------------------+------------------+------------------------+------------------+

| Q65UNOFF1 | crude | wt | 95% CI | 90% CI | 99% CI | MH Summ | test for | p-value |

| | OR | OR | wt OR | wt OR | wt OR | Chi Sq | interaction | |

+------------+--------+------+-------------+-------------+-------------+----------+-------------+----------+

| Q73GIFT1 | 4.72 | 4.04 | 2.72 - 6.00 | 2.90 - 5.63 | 2.40 - 6.80 | 47.63 | 6.0092 | 0.1112 |

+------------+--------+------+-------------+-------------+-------------+----------+-------------+----------+

| AREA1 | 2.34 | 2.02 | 1.16 - 3.52 | 1.27 - 3.22 | 0.97 - 4.19 | 6.14 | 4.0619 | 0.2549 |

+------------+--------+------+-------------+-------------+-------------+----------+-------------+----------+

| Q37EDUC1 | 2.91 | 2.53 | 1.69 - 3.80 | 1.80 - 3.56 | 1.49 - 4.32 | 20.12 | 6.7454 | 0.0805 |

+------------+--------+------+-------------+-------------+-------------+----------+-------------+----------+

Lithuania

+------------+--------+------+-------------+-------------+-------------+----------+-------------+----------+

| Q65UNOFF1 | crude | wt | 95% CI | 90% CI | 99% CI | MH Summ | test for | p-value |

| | OR | OR | wt OR | wt OR | wt OR | Chi Sq | interaction | |

+------------+--------+------+-------------+-------------+-------------+----------+-------------+----------+

| Q73GIFT1 | 3.59 | 3.28 | 2.36 - 4.56 | 2.49 - 4.32 | 2.13 - 5.05 | 50.30 | 3.4043 | 0.8453 |

+------------+--------+------+-------------+-------------+-------------+----------+-------------+----------+

| Q36AGE50 | 1.46 | 1.39 | 1.02 - 1.89 | 1.07 - 1.80 | 0.92 - 2.08 | 4.32 | 4.7644 | 0.6887 |

+------------+--------+------+-------------+-------------+-------------+----------+-------------+----------+

| Q79SUFF1 | 1.62 | 1.46 | 1.01 - 2.10 | 1.08 - 1.98 | 0.90 - 2.35 | 4.15 | 3.0212 | 0.8830 |

+------------+--------+------+-------------+-------------+-------------+----------+-------------+----------+

| Q37EDUC2 | 2.31 | 2.10 | 1.55 - 2.86 | 1.62 - 2.72 | 1.40 - 3.15 | 22.46 | 2.4331 | 0.9321 |

+------------+--------+------+-------------+-------------+-------------+----------+-------------+----------+

Table 9. Variables related to willingness to pay to avoid a waiting list.

Logistic regression models on which table 9 in the main text is based:

Estonia

+------------+--------+------+-------------+-------------+-------------+----------+-------------+----------+

| Q27LIST1 | crude | wt | 95% CI | 90% CI | 99% CI | MH Summ | test for | p-value |

| | OR | OR | wt OR | wt OR | wt OR | Chi Sq | interaction | |

+------------+--------+------+-------------+-------------+-------------+----------+-------------+----------+

| Q2RESPAGE1 | 5.27 | 4.75 | 4.03 - 5.59 | 4.14 - 5.45 | 3.83 - 5.89 | 346.40 | 5.1254 | 0.1628 |

+------------+--------+------+-------------+-------------+-------------+----------+-------------+----------+

| Q79SUFF1 | 1.52 | 1.45 | 1.22 - 1.72 | 1.26 - 1.68 | 1.16 - 1.82 | 17.99 | 16.6446 | 0.0008 |

+------------+--------+------+-------------+-------------+-------------+----------+-------------+----------+

| Q3WINEDU1 | 0.43 | 0.60 | 0.51 - 0.71 | 0.52 - 0.69 | 0.48 - 0.74 | 37.18 | 9.8141 | 0.0202 |

+------------+--------+------+-------------+-------------+-------------+----------+-------------+----------+

Latvia

+------------+--------+------+-------------+-------------+-------------+----------+-------------+----------+

| q27list2 | crude | wt | 95% CI | 90% CI | 99% CI | MH Summ | test for | p-value |

| | OR | OR | wt OR | wt OR | wt OR | Chi Sq | interaction | |

+------------+--------+------+-------------+-------------+-------------+----------+-------------+----------+

| Q2RESPAGE1 | 5.16 | 4.73 | 4.02 - 5.55 | 4.13 - 5.41 | 3.83 - 5.84 | 357.78 | 23.6196 | 0.0718 |

+------------+--------+------+-------------+-------------+-------------+----------+-------------+----------+

| Q2RESPSEX1 | 1.77 | 1.49 | 1.24 - 1.80 | 1.28 - 1.75 | 1.17 - 1.91 | 17.59 | 12.7929 | 0.6183 |

+------------+--------+------+-------------+-------------+-------------+----------+-------------+----------+

| Q79SUFF1 | 1.95 | 1.94 | 1.58 - 2.37 | 1.63 - 2.30 | 1.48 - 2.53 | 40.82 | 18.1954 | 0.2525 |

+------------+--------+------+-------------+-------------+-------------+----------+-------------+----------+

| Q4LANG1 | 0.83 | 0.80 | 0.67 - 0.95 | 0.69 - 0.93 | 0.63 - 1.01 | 6.30 | 19.7009 | 0.1837 |

+------------+--------+------+-------------+-------------+-------------+----------+-------------+----------+

| Q3WINEDU1 | 2.05 | 1.60 | 1.36 - 1.88 | 1.40 - 1.83 | 1.29 - 1.98 | 32.61 | 26.1506 | 0.0365 |

+------------+--------+------+-------------+-------------+-------------+----------+-------------+----------+

Lithuania

+------------+--------+------+-------------+-------------+-------------+----------+-------------+----------+

| Q27LIST1 | crude | wt | 95% CI | 90% CI | 99% CI | MH Summ | test for | p-value |

| | OR | OR | wt OR | wt OR | wt OR | Chi Sq | interaction | |

+------------+--------+------+-------------+-------------+-------------+----------+-------------+----------+

| Q2RESPAGE1 | 3.69 | 3.26 | 2.78 - 3.82 | 2.86 - 3.73 | 2.65 - 4.02 | 214.16 | 7.0676 | 0.4219 |

+------------+--------+------+-------------+-------------+-------------+----------+-------------+----------+

| Q79SUFF1 | 1.51 | 1.52 | 1.26 - 1.85 | 1.30 - 1.79 | 1.18 - 1.96 | 18.49 | 16.2536 | 0.0229 |

+------------+--------+------+-------------+-------------+-------------+----------+-------------+----------+

| Q4LANG1 | 1.42 | 1.48 | 1.09 - 2.00 | 1.15 - 1.91 | 0.99 - 2.20 | 6.40 | 10.0412 | 0.1863 |

+------------+--------+------+-------------+-------------+-------------+----------+-------------+----------+

| Q3WINEDU1 | 0.35 | 0.60 | 0.47 - 0.76 | 0.49 - 0.73 | 0.44 - 0.82 | 17.81 | 8.6618 | 0.2779 |

+------------+--------+------+-------------+-------------+-------------+----------+-------------+----------+

**Key to variable names**

Q34RATE1 Household rating of corruption in government health services

1=high 2= neither/nor -low

Q4LANG1 The main language in the household

1= National language 2=other

Q20KNOW1 Respondent feels they have all the information they need about health care entitlements

1= yes 2=no

Q79SUFF1 Household has sufficient income to meet expenditure needs

1= yes 2=no

Q3WINEDU1 Breadwinner’s level of education

1=less than basic 2= basic or more

Q2RESPSEX1 Sex of the respondent

1= male 2=female

Q2RESPAGE1 Age of the respondent

1=less than 30 2= 30 or more

Q65UNOFF1 Respondents reporting making an unofficial payment

1= yes 2=no

Q73GIFT1 Respondents who gave any gift for care received

1=yes 2=no

Q36AGE50 Age of health care user

1= less than 50 2= 50 or more

AREA1 Urban or rural community

1= urban 2=rural

Q37EDUC1 Education level of health care user

1= less than basic 2=basic or more

Q27LIST Willing to pay to avoid a waiting list for surgery or other hospital treatment

1=yes 2=no

**List of variables used in the initial saturated models**

Sex of respondent

Age of the respondent

Urban or rural community

Household has sufficient income to meet expenditure needs

Main language used in the household

Education of the main breadwinner

Education level of the health care user

Know enough about health care entitlements
